# Adaptive Synchronization of Fractional-Order Complex-Valued Neural Networks with Discrete and Distributed Delays

**DOI:** 10.3390/e20020124

**Published:** 2018-02-13

**Authors:** Li Li, Zhen Wang, Junwei Lu, Yuxia Li

**Affiliations:** 1 College of Mathematics and Systems Science, Shandong University of Science and Technology, Qingdao 266590, China; 2 College of Electrical Engineering and Automation, Shandong University of Science and Technology, Qingdao 266590, China; 3 School of Electrical and Automation Engineering, Nanjing Normal University, Nanjing 210023, China

**Keywords:** complex-valued information, fractional-order, neural networks, delay, synchronization

## Abstract

In this paper, the synchronization problem of fractional-order complex-valued neural networks with discrete and distributed delays is investigated. Based on the adaptive control and Lyapunov function theory, some sufficient conditions are derived to ensure the states of two fractional-order complex-valued neural networks with discrete and distributed delays achieve complete synchronization rapidly. Finally, numerical simulations are given to illustrate the effectiveness and feasibility of the theoretical results.

## 1. Introduction

The complex-valued neural networks (CVNNs) are the networks that deal with complex-valued information by using complex-valued parameters and variables [1]. They have more different and complicated properties than the real-valued neural networks (RVNNs). CVNNs possess new capabilities and higher performance, which makes it possible to solve some problems that cannot be solved by their real-valued counterparts [2,3]. Actually, most of the applications of neural networks (NNs) involve complex information [4,5]. Therefore, it is of great significance to study the dynamical properties of CVNNs [6,7,8,9,10,11,12,13,14]. In recent years, CVNNs have received considerable attention due to their widespread applications in signal processing, quantum waves, remote sensing, optoelectronics, filtering, electromagnetic, speech synthesis, and so on [15,16].

Nowadays, fractional calculus has become a hot topic and many applications have been found in the fields of physics and engineering [17,18,19,20]. Fractional calculus is the generalization of classic calculus, which deals with derivatives and integrals of arbitrary order. Many real world objects can be described by the fractional-order models, such as dielectric polarization, electromagnetic waves, entropy and information [21,22,23,24]. The main advantage of fractional-order models in comparison with their integer-order counterparts is that fractional derivatives provide an excellent instrument in the description of memory and hereditary properties of various materials and process [25,26]. In addition, fractional-order models are characterized by infinite memory [27,28,29]. Thus, fractional-order NNs (FNNs) are more effective in information processing than integer-order NNs [30]. In recent years, the dynamics of FNNs has been investigated by many researchers and some interesting results have been achieved [31,32,33,34,35]. In [31], fractional-order cellular NNs have been presented and hyperchaotic attractors have been displayed. In [32,33,34], chaos control and synchronization of FNNs were investigated by using the Laplace transformation or Lyapunov method. In [35], the dynamics, including stability and multistability, of FNNs with the ring or hub structure has been investigated.

As is well known, time delays may affect and even destroy the dynamics of NNs [36,37,38,39,40]. Due to the signals propagation through the links and the frequently delayed couplings in biological NNs, time delay unavoidably exists in NNs [41,42]. In particularly, since the presence of an amount of parallel pathways with a variety of node sizes and lengths, NNs usually have spatial extent. Thus, there will be a distribution of propagation delays. Therefore, the study of fractional-order complex-valued neural networks (FCVNNs) with time delays is of both theoretical and practical significance. At present, the investigations of FCVNNs with discrete time delay have achieved many remarkable results [43,44,45,46]. For example, authors in [43,44,45] discussed the problem of stability of FCVNNs with time delays. Finite-time stability of fractional-order complex-valued memristor-based NNs with time delays has been intensively investigated in [46]. However, the dynamics of FNNs with distributed delay is even more complicated. Very recently, study concerning FNNs with distributed delay has become an active research topic. Many researchers have devoted to the investigation of FNNs with distributed delay and some results have been derived [47]. In [47], two sufficient conditions, which guarantee the asymptotic stability of the Riemann-Liouville FNNs with discrete and distributed delays, have been derived in terms of LMI.

So far, the synchronization of the integer-order CVNNs with time delays has been intensively studied by applying various control schemes [48,49,50,51]. However, using integer-order CVNNs with time delays to model real systems with memory and hereditary properties are inadequate in contrast with FCVNNs with discrete time delay [52]. To the best of our knowledge, few investigations have been devoted to the control and information synchronization of FCVNNs with time delays in spite of its practical significance. In [36,53], the problem of synchronization of FCVNNs with discrete time delays is analyzed and sufficient conditions are provided. On the other hand, adaptive control, as an efficient control method, has been designed and successfully applied to fractional order neural networks [34,54]. Motivated by the above discussions, this paper is devoted to investigating the problem of information synchronization of FCVNNs with discrete and distributed delays. An adaptive controller is designed to synchronize two FCVNNs with discrete and distributed delays. Based on adaptive control and Lyapunov stability theory, some sufficient conditions are derived to ensure that two FCVNNs with discrete and distributed delays can achieve information synchronization rapidly.

This paper is organized as follows. In Section 2, some definitions in the fractional-order calculus and some lemmas, which will be used later, are introduced. The adaptive controller is designed in Section 3. In Section 4, a numerical example is given to illustrate the effectiveness of the main results. Finally, conclusions are drawn in Section 5.

## 2. Preliminaries

There are several different definitions for fractional derivatives. Three of the most frequently used definitions are the Riemann-Liouville definition, the Grünwald-Letnikov definition and the Caputo definition. Since the initial conditions for fractional differential equations with Caputo derivatives take on the same form as for integer-order differential equations, we choose the Caputo definition in this paper.

**Definition 1** **([17]).** *The fractional integral of order α for a function f is defined as*

(1) $$I^{\alpha }f\left(t\right)=\frac{1}{\Gamma \left(\alpha \right)}\int_{0}^{t}{\left(t-s\right)}^{\alpha -1}f\left(s\right)\;ds,$$

*where t≥0 and α>0, Γ(·) is the Gamma function defined as $\Gamma \left(z\right)=\int_{0}^{\infty }t^{z-1}e^{-t}dt$.*

**Definition 2** **([17]).** *The Caputo fractional derivative of order α for a function f is defined as follows:*

(2) $$D^{\alpha }f\left(t\right)=\frac{1}{\Gamma \left(n-\alpha \right)}\int_{0}^{t}{\left(t-s\right)}^{n-\alpha -1}f^{\left(n\right)}\left(s\right)\;ds,$$

*where n is the positive integer such that n−1<α<n.*

**Lemma 1** **([55]).** *Let x(t)∈R be a continuous and differentiable function, then for any time instant t≥0,*

$$\frac{1}{2}D^{\alpha }x^{2}\left(t\right)\leq x\left(t\right)D^{\alpha }x\left(t\right),\;\;\;\forall \alpha \in \left(0,1\right).$$

**Lemma 2** **([56]).** *If $e\left(t\right)\in C^{1}\left(\left[0,+\infty \right],R\right)$ denotes a continuously differentiable function, for any α∈(0,1), the following inequality holds almost everywhere:*

$$D^{\alpha }\left|e\left(t\right)\right|\leq sgn\left(e\left(t\right)\right)D^{\alpha }e\left(t\right).$$

Consider a simplified CVNN with discrete and distributed delays as the drive system, which is described by

(3) $$\begin{array}{c} D^{\alpha }x_{1}\left(t\right)=-a_{1}x_{1}\left(t\right)+b_{11}f\left(\int_{-\infty }^{t}F\left(t-s\right)x_{1}\left(s\right)\;ds\right)+b_{12}g\left(x_{2}\left(t-\tau \right)\right)+I_{1}\left(t\right),\\ D^{\alpha }x_{2}\left(t\right)=-a_{2}x_{2}\left(t\right)+b_{21}f\left(x_{2}\left(t\right)\right)+b_{22}g\left(x_{1}\left(t-\tau \right)\right)+I_{2}\left(t\right), \end{array}$$

where 0<α<1 denotes the fractional order, $x_{i}\left(t\right)$ (i=1,2) is the state of the *i*th neuron at time *t*, $a_{i}>0$ (i=1,2), $b_{ij}$ (i,j=1,2) are complex constants, τ is the discrete time delay, $I_{i}\left(t\right)$ (i=1,2) are the external inputs, f(·) and g(·) denote the complex-valued activation functions, and F(·) denotes non-negative bounded delay kernel defined on [0,+∞) which reflects the influence of the past states on the current dynamics.

In general, the kernel F(s) is taken as the following form:

(4) $$F\left(s\right)=a_{3}e^{-a_{3}s},\;\left(a_{3}>0,s\geq 0\right),$$

where $a_{3}$ reflects the mean delay of the kernel.

For convenience, a new variable $x_{3}\left(t\right)$ is introduced and defined as:

(5) $$x_{3}\left(t\right)=\int_{-\infty }^{t}F\left(t-s\right)x_{1}\left(s\right)\;ds.$$

Then, one can rewrite the drive system as

(6) $$\begin{array}{c} D^{\alpha }x_{1}\left(t\right)=-a_{1}x_{1}\left(t\right)+b_{11}f\left(x_{3}\left(t\right)\right)+b_{12}g\left(x_{2}\left(t-\tau \right)\right)+I_{1}\left(t\right),\\ D^{\alpha }x_{2}\left(t\right)=-a_{2}x_{2}\left(t\right)+b_{21}f\left(x_{2}\left(t\right)\right)+b_{22}g\left(x_{1}\left(t-\tau \right)\right)+I_{2}\left(t\right),\\ x_{3}^{\prime }\left(t\right)=-a_{3}x_{3}\left(t\right)+a_{3}x_{1}\left(t\right). \end{array}$$

where $x_{i}\left(t\right)=u_{i}\left(t\right)+iv_{i}\left(t\right)$ (i=1,2,3), $u_{i}\left(t\right)=Re\left(x_{i}\left(t\right)\right)$, $v_{i}\left(t\right)=Im\left(x_{i}\left(t\right)\right)$.

Similarly, the response system is defined as follows:

(7) $$\begin{array}{c} D^{\alpha }y_{1}\left(t\right)=-a_{1}y_{1}\left(t\right)+b_{11}f\left(y_{3}\left(t\right)\right)+b_{12}g\left(y_{2}\left(t-\tau \right)\right)+I_{1}\left(t\right)+U_{1}\left(t\right),\\ D^{\alpha }y_{2}\left(t\right)=-a_{2}y_{2}\left(t\right)+b_{21}f\left(y_{2}\left(t\right)\right)+b_{22}g\left(y_{1}\left(t-\tau \right)\right)+I_{2}\left(t\right)+U_{2}\left(t\right),\\ y_{3}^{\prime }\left(t\right)=-a_{3}y_{3}\left(t\right)+a_{3}y_{1}\left(t\right). \end{array}$$

where $U_{i}\left(t\right)$ (i=1,2) are the control inputs to be designed later, $y_{i}\left(t\right)={\bar{u}}_{i}\left(t\right)+i{\bar{v}}_{i}\left(t\right)$ (i=1,2,3), ${\bar{u}}_{i}\left(t\right)=Re\left(y_{i}\left(t\right)\right)$, ${\bar{v}}_{i}\left(t\right)=Im\left(y_{i}\left(t\right)\right)$.

To obtain the main results, one makes the following assumption.

**Assumption** **1.** *Let $u_{\tau }=u\left(t-\tau \right)$, $v_{\tau }=v\left(t-\tau \right)$, x=u+iv, $y=\bar{u}+i\bar{v}$. f(x) and g(x(t−τ)) can be expressed by separating into its real and imaginary parts as*

$$f\left(x\right)=f^{R}\left(u,v\right)+if^{I}\left(u,v\right),g\left(x\left(t-\tau \right)\right)=g^{R}\left(u_{\tau },v_{\tau }\right)+ig^{I}\left(u_{\tau },v_{\tau }\right).$$

**Assumption** **2.** *The partial derivatives of $f^{R}\left(u,v\right)$, $f^{I}\left(u,v\right)$, $g^{R}\left(u_{\tau },v_{\tau }\right)$ and $g^{I}\left(u_{\tau },v_{\tau }\right)$ with respect to u, v, exist and are continuous and bounded. In addition, $f^{R}\left(\cdot ,\cdot \right):R^{2}\to R$, $f^{I}\left(\cdot ,\cdot \right):R^{2}\to R$, $g^{R}\left(\cdot ,\cdot \right):R^{2}\to R$ and $g^{I}\left(\cdot ,\cdot \right):R^{2}\to R$ satisfy*

$$\begin{array}{c} \left|f^{R}\left(\bar{u},\bar{v}\right)-f^{R}\left(u,v\right)\right|\leq \lambda^{RR}|\bar{u}-u|+\lambda^{RI}\left|\bar{v}-v\right|,\\ \left|f^{I}\left(\bar{u},\bar{v}\right)-f^{I}\left(u,v\right)\right|\leq \lambda^{IR}|\bar{u}-u|+\lambda^{II}\left|\bar{v}-v\right|,\\ \left|g^{R}\left({\bar{u}}_{\tau },{\bar{v}}_{\tau }\right)-g^{R}\left(u_{\tau },v_{\tau }\right)\right|\leq \mu^{RR}|{\bar{u}}_{\tau }-u_{\tau }|+\mu^{RI}\left|{\bar{v}}_{\tau }-v_{\tau }\right|,\\ \left|g^{I}\left({\bar{u}}_{\tau },{\bar{v}}_{\tau }\right)-g^{I}\left(u_{\tau },v_{\tau }\right)\right|\leq \mu^{IR}|{\bar{u}}_{\tau }-u_{\tau }|+\mu^{II}\left|{\bar{v}}_{\tau }-v_{\tau }\right|, \end{array}$$

*where*

$$\begin{array}{c} \left|\frac{\partial f^{R}}{\partial u}\right|\leq \lambda^{RR},\;\left|\frac{\partial f^{R}}{\partial v}\right|\leq \lambda^{RI},\;\left|\frac{\partial f^{I}}{\partial u}\right|\leq \lambda^{IR},\;\left|\frac{\partial f^{I}}{\partial v}\right|\leq \lambda^{II},\\ \left|\frac{\partial g^{R}}{\partial u}\right|\leq \mu^{RR},\;\left|\frac{\partial g^{R}}{\partial v}\right|\leq \mu^{RI},\;\left|\frac{\partial g^{I}}{\partial u}\right|\leq \mu^{IR},\;\left|\frac{\partial g^{I}}{\partial v}\right|\leq \mu^{II}. \end{array}$$

From Assumptions 1 and 2, FCVNNs (6) and (7) can be separated into its real and imaginary parts, respectively. Then, one has

(8) $$\begin{array}{c} D^{\alpha }u_{1}\left(t\right)=-a_{1}u_{1}\left(t\right)+b_{11}^{R}f^{R}\left(u_{3}\left(t\right),v_{3}\left(t\right)\right)-b_{11}^{I}f^{I}\left(u_{3}\left(t\right),v_{3}\left(t\right)\right)+b_{12}^{R}g^{R}\left(u_{2}\left(t-\tau \right),v_{2}\left(t-\tau \right)\right)\\ \;\;-b_{12}^{I}g^{I}\left(u_{2}\left(t-\tau \right),v_{2}\left(t-\tau \right)\right)+I_{1}^{R}\left(t\right),\\ D^{\alpha }u_{2}\left(t\right)=-a_{2}u_{2}\left(t\right)+b_{21}^{R}f^{R}\left(u_{2}\left(t\right),v_{2}\left(t\right)\right)-b_{21}^{I}f^{I}\left(u_{2}\left(t\right),v_{2}\left(t\right)\right)+b_{22}^{R}g^{R}\left(u_{1}\left(t-\tau \right),v_{1}\left(t-\tau \right)\right)\\ \;\;-b_{22}^{I}g^{I}\left(u_{1}\left(t-\tau \right),v_{1}\left(t-\tau \right)\right)+I_{2}^{R}\left(t\right),\\ u_{3}^{\prime }\left(t\right)=-a_{3}u_{3}\left(t\right)+a_{3}u_{1}\left(t\right),\\ D^{\alpha }v_{1}\left(t\right)=-a_{1}v_{1}\left(t\right)+b_{11}^{R}f^{I}\left(u_{3}\left(t\right),v_{3}\left(t\right)\right)+b_{11}^{I}f^{R}\left(u_{3}\left(t\right),v_{3}\left(t\right)\right)+b_{12}^{R}g^{I}\left(u_{2}\left(t-\tau \right),v_{2}\left(t-\tau \right)\right)\\ \;\;+b_{12}^{I}g^{R}\left(u_{2}\left(t-\tau \right),v_{2}\left(t-\tau \right)\right)+I_{1}^{I}\left(t\right),\\ D^{\alpha }v_{2}\left(t\right)=-a_{2}v_{2}\left(t\right)+b_{21}^{R}f^{I}\left(u_{2}\left(t\right),v_{2}\left(t\right)\right)+b_{21}^{I}f^{R}\left(u_{2}\left(t\right),v_{2}\left(t\right)\right)+b_{22}^{R}g^{I}\left(u_{1}\left(t-\tau \right),v_{1}\left(t-\tau \right)\right)\\ \;\;+b_{22}^{I}g^{R}\left(u_{1}\left(t-\tau \right),v_{1}\left(t-\tau \right)\right)+I_{2}^{I}\left(t\right),\\ v_{3}^{\prime }\left(t\right)=-a_{3}v_{3}\left(t\right)+a_{3}v_{1}\left(t\right). \end{array}$$

and

(9) $$\begin{array}{c} D^{\alpha }{\bar{u}}_{1}\left(t\right)=-a_{1}{\bar{u}}_{1}\left(t\right)+b_{11}^{R}f^{R}\left({\bar{u}}_{3}\left(t\right),{\bar{v}}_{3}\left(t\right)\right)-b_{11}^{I}f^{I}\left({\bar{u}}_{3}\left(t\right),{\bar{v}}_{3}\left(t\right)\right)+b_{12}^{R}g^{R}\left({\bar{u}}_{2}\left(t-\tau \right),{\bar{v}}_{2}\left(t-\tau \right)\right)\\ \;\;-b_{12}^{I}g^{I}\left({\bar{u}}_{2}\left(t-\tau \right),{\bar{v}}_{2}\left(t-\tau \right)\right)+I_{1}^{R}\left(t\right)+U_{1}^{R}\left(t\right),\\ D^{\alpha }{\bar{u}}_{2}\left(t\right)=-a_{2}{\bar{u}}_{2}\left(t\right)+b_{21}^{R}f^{R}\left({\bar{u}}_{2}\left(t\right),{\bar{v}}_{2}\left(t\right)\right)-b_{21}^{I}f^{I}\left({\bar{u}}_{2}\left(t\right),{\bar{v}}_{2}\left(t\right)\right)+b_{22}^{R}g^{R}\left({\bar{u}}_{1}\left(t-\tau \right),{\bar{v}}_{1}\left(t-\tau \right)\right)\\ \;\;-b_{22}^{I}g^{I}\left({\bar{u}}_{1}\left(t-\tau \right),{\bar{v}}_{1}\left(t-\tau \right)\right)+I_{2}^{R}\left(t\right)+U_{2}^{R}\left(t\right),\\ {\bar{u}}_{3}^{\prime }\left(t\right)=-a_{3}{\bar{u}}_{3}\left(t\right)+a_{3}{\bar{u}}_{1}\left(t\right),\\ D^{\alpha }{\bar{v}}_{1}\left(t\right)=-a_{1}{\bar{v}}_{1}\left(t\right)+b_{11}^{R}f^{I}\left({\bar{u}}_{3}\left(t\right),{\bar{v}}_{3}\left(t\right)\right)+b_{11}^{I}f^{R}\left({\bar{u}}_{3}\left(t\right),{\bar{v}}_{3}\left(t\right)\right)+b_{12}^{R}g^{I}\left({\bar{u}}_{2}\left(t-\tau \right),{\bar{v}}_{2}\left(t-\tau \right)\right)\\ \;\;+b_{12}^{I}g^{R}\left({\bar{u}}_{2}\left(t-\tau \right),{\bar{v}}_{2}\left(t-\tau \right)\right)+I_{1}^{I}\left(t\right)+U_{1}^{I}\left(t\right),\\ D^{\alpha }{\bar{v}}_{2}\left(t\right)=-a_{2}{\bar{v}}_{2}\left(t\right)+b_{21}^{R}f^{I}\left({\bar{u}}_{2}\left(t\right),{\bar{v}}_{2}\left(t\right)\right)+b_{21}^{I}f^{R}\left({\bar{u}}_{2}\left(t\right),{\bar{v}}_{2}\left(t\right)\right)+b_{22}^{R}g^{I}\left({\bar{u}}_{1}\left(t-\tau \right),{\bar{v}}_{1}\left(t-\tau \right)\right)\\ \;\;+b_{22}^{I}g^{R}\left({\bar{u}}_{1}\left(t-\tau \right),{\bar{v}}_{1}\left(t-\tau \right)\right)+I_{2}^{I}\left(t\right)+U_{2}^{I}\left(t\right),\\ {\bar{v}}_{3}^{\prime }\left(t\right)=-a_{3}{\bar{v}}_{3}\left(t\right)+a_{3}{\bar{v}}_{1}\left(t\right). \end{array}$$

where $f^{R}\left(\cdot ,\cdot \right)=Re\left(f\left(\cdot ,\cdot \right)\right)$, $f^{I}\left(\cdot ,\cdot \right)=Im\left(f\left(\cdot ,\cdot \right)\right)$, $g^{R}\left(\cdot ,\cdot \right)=Re\left(g\left(\cdot ,\cdot \right)\right)$, $g^{I}\left(\cdot ,\cdot \right)=Im\left(g\left(\cdot ,\cdot \right)\right)$, $b_{ij}^{R}=Re\left(b_{ij}\right)$, $b_{ij}^{I}=Im\left(b_{ij}\right)$, $I_{i}^{R}\left(t\right)=Re\left(I_{i}\left(t\right)\right)$, $I_{i}^{I}\left(t\right)=Im\left(I_{i}\left(t\right)\right)$, $U_{i}^{R}\left(t\right)=Re\left(U_{i}\left(t\right)\right)$, $U_{i}^{I}\left(t\right)=Im\left(U_{i}\left(t\right)\right)$.

## 3. Main Results

In this section, some sufficient conditions for the information synchronization of FCVNNs with discrete and distributed delays are derived.

Let $e_{i}\left(t\right)=y_{i}\left(t\right)-x_{i}\left(t\right)=e_{i}^{u}\left(t\right)+ie_{i}^{v}\left(t\right)$ (i=1,2,3). Subtracting the drive system (8) from the response system (9), one obtains the error system as follows:

(10) $$\begin{array}{c} D^{\alpha }e_{1}^{u}\left(t\right)=-a_{1}e_{1}^{u}\left(t\right)+b_{11}^{R}\left[f^{R}\left({\bar{u}}_{3}\left(t\right),{\bar{v}}_{3}\left(t\right)\right)-f^{R}\left(u_{3}\left(t\right),v_{3}\left(t\right)\right)\right]-b_{11}^{I}\left[f^{I}\left({\bar{u}}_{3}\left(t\right),{\bar{v}}_{3}\left(t\right)\right)-f^{I}\left(u_{3}\left(t\right),v_{3}\left(t\right)\right)\right]\\ \;\;+b_{12}^{R}\left[g^{R}\left({\bar{u}}_{2}\left(t-\tau \right),{\bar{v}}_{2}\left(t-\tau \right)\right)-g^{R}\left(u_{2}\left(t-\tau \right),v_{2}\left(t-\tau \right)\right)\right]-b_{12}^{I}[g^{I}\left({\bar{u}}_{2}\left(t-\tau \right),{\bar{v}}_{2}\left(t-\tau \right)\right)\\ \;\;-g^{I}\left(u_{2}\left(t-\tau \right),v_{2}\left(t-\tau \right)\right)]+U_{1}^{R}\left(t\right),\\ D^{\alpha }e_{2}^{u}\left(t\right)=-a_{2}e_{2}^{u}\left(t\right)+b_{21}^{R}\left[f^{R}\left({\bar{u}}_{2}\left(t\right),{\bar{v}}_{2}\left(t\right)\right)-f^{R}\left(u_{2}\left(t\right),v_{2}\left(t\right)\right)\right]-b_{21}^{I}\left[f^{I}\left({\bar{u}}_{2}\left(t\right),{\bar{v}}_{2}\left(t\right)\right)-f^{I}\left(u_{2}\left(t\right),v_{2}\left(t\right)\right)\right]\\ \;\;+b_{22}^{R}\left[g^{R}\left({\bar{u}}_{1}\left(t-\tau \right),{\bar{v}}_{1}\left(t-\tau \right)\right)-g^{R}\left(u_{1}\left(t-\tau \right),v_{1}\left(t-\tau \right)\right)\right]-b_{22}^{I}[g^{I}\left({\bar{u}}_{1}\left(t-\tau \right),{\bar{v}}_{1}\left(t-\tau \right)\right)\\ \;\;-g^{I}\left(u_{1}\left(t-\tau \right),v_{1}\left(t-\tau \right)\right)]+U_{2}^{R}\left(t\right),\\ {\left[e_{3}^{u}\left(t\right)\right]}^{\prime }=-a_{3}e_{3}^{u}\left(t\right)+a_{3}e_{1}^{u}\left(t\right),\\ D^{\alpha }e_{1}^{v}\left(t\right)=-a_{1}e_{1}^{v}\left(t\right)+b_{11}^{R}\left[f^{I}\left({\bar{u}}_{3}\left(t\right),{\bar{v}}_{3}\left(t\right)\right)-f^{I}\left(u_{3}\left(t\right),v_{3}\left(t\right)\right)\right]+b_{11}^{I}\left[f^{R}\left({\bar{u}}_{3}\left(t\right),{\bar{v}}_{3}\left(t\right)\right)-f^{R}\left(u_{3}\left(t\right),v_{3}\left(t\right)\right)\right]\\ \;\;+b_{12}^{R}\left[g^{I}\left({\bar{u}}_{2}\left(t-\tau \right),{\bar{v}}_{2}\left(t-\tau \right)\right)-g^{I}\left(u_{2}\left(t-\tau \right),v_{2}\left(t-\tau \right)\right)\right]+b_{12}^{I}[g^{R}\left({\bar{u}}_{2}\left(t-\tau \right),{\bar{v}}_{2}\left(t-\tau \right)\right)\\ \;\;-g^{R}\left(u_{2}\left(t-\tau \right),v_{2}\left(t-\tau \right)\right)]+U_{1}^{I}\left(t\right),\\ D^{\alpha }e_{2}^{v}\left(t\right)=-a_{2}e_{2}^{v}\left(t\right)+b_{21}^{R}\left[f^{I}\left({\bar{u}}_{2}\left(t\right),{\bar{v}}_{2}\left(t\right)\right)-f^{I}\left(u_{2}\left(t\right),v_{2}\left(t\right)\right)\right]+b_{21}^{I}\left[f^{R}\left({\bar{u}}_{2}\left(t\right),{\bar{v}}_{2}\left(t\right)\right)-f^{R}\left(u_{2}\left(t\right),v_{2}\left(t\right)\right)\right]\\ \;\;+b_{22}^{R}\left[g^{I}\left({\bar{u}}_{1}\left(t-\tau \right),{\bar{v}}_{1}\left(t-\tau \right)\right)-g^{I}\left(u_{1}\left(t-\tau \right),v_{1}\left(t-\tau \right)\right)\right]+b_{22}^{I}[g^{R}\left({\bar{u}}_{1}\left(t-\tau \right),{\bar{v}}_{1}\left(t-\tau \right)\right)\\ \;\;-g^{R}\left(u_{1}\left(t-\tau \right),v_{1}\left(t-\tau \right)\right)]+U_{2}^{I}\left(t\right),\\ {\left[e_{3}^{v}\left(t\right)\right]}^{\prime }=-a_{3}e_{3}^{v}\left(t\right)+a_{3}e_{1}^{v}\left(t\right). \end{array}$$

Design the following control input

(11) $$\left\{\begin{array}{c} U_{1}^{R}\left(t\right)=-d_{1}\left(t\right)e_{1}^{u}\left(t\right)-sgn\left(e_{1}^{u}\left(t\right)\right)\eta_{1}\left(t\right)\left|e_{1}^{u}\left(t-\tau \right)\right|-w_{1}\left(t\right)e_{3}^{u}\left(t\right),\\ U_{2}^{R}\left(t\right)=-d_{2}\left(t\right)e_{2}^{u}\left(t\right)-sgn\left(e_{2}^{u}\left(t\right)\right)\eta_{2}\left(t\right)\left|e_{2}^{u}\left(t-\tau \right)\right|,\\ U_{1}^{I}\left(t\right)=-p_{1}\left(t\right)e_{1}^{v}\left(t\right)-sgn\left(e_{1}^{v}\left(t\right)\right)\theta_{1}\left(t\right)\left|e_{1}^{v}\left(t-\tau \right)\right|-w_{2}\left(t\right)e_{3}^{v}\left(t\right),\\ U_{2}^{I}\left(t\right)=-p_{2}\left(t\right)e_{2}^{v}\left(t\right)-sgn\left(e_{2}^{v}\left(t\right)\right)\theta_{2}\left(t\right)\left|e_{2}^{v}\left(t-\tau \right)\right|,\\ D^{\alpha }d_{i}\left(t\right)=k_{i}\left|e_{i}^{u}\left(t\right)\right|,\;\left(i=1,2\right),\\ D^{\alpha }\eta_{i}\left(t\right)=m_{i}\left|e_{i}^{u}\left(t-\tau \right)\right|,\;\left(i=1,2\right),\\ D^{\alpha }p_{i}\left(t\right)=l_{i}\left|e_{i}^{v}\left(t\right)\right|,\;\left(i=1,2\right),\\ D^{\alpha }\theta_{i}\left(t\right)=n_{i}\left|e_{i}^{v}\left(t-\tau \right)\right|,\;\left(i=1,2\right),\\ D^{\alpha }w_{1}\left(t\right)=q_{1}\left|e_{3}^{u}\left(t\right)\right|,\\ D^{\alpha }w_{2}\left(t\right)=q_{2}\left|e_{3}^{v}\left(t\right)\right|, \end{array}\right.$$

where $d_{i}\left(t\right)$, $\eta_{i}\left(t\right)$, $p_{i}\left(t\right)$, $\theta_{i}\left(t\right)$ and $w_{i}\left(t\right)$ are adjustable parameters, $k_{i}$, $m_{i}$, $l_{i}$, $q_{i}$ and $n_{i}$ are arbitrary positive constants. When $e_{i}^{u}\left(t\right)\to 0$ and $e_{i}^{v}\left(t\right)\to 0$ (i=1,2), the drive system (6) and the response system (7) achieve the information synchronization, which can be ensured by the following theorem.

**Theorem** **1.** Under Assumptions 1 and 2, the drive system *(6)* and the response system *(7)* can achieve globally asymptotically synchronized with the controller *(11)*.

**Proof.**  Suppose that $x_{i}\left(t\right)=u_{i}\left(t\right)+iv_{i}\left(t\right)$ and $y_{i}\left(t\right)={\bar{u}}_{i}\left(t\right)+i{\bar{v}}_{i}\left(t\right)$ are any solution of systems (6) and (7) with different initial values. Let

$$\begin{array}{c} V_{1}\left(t\right)=\sum_{i=1}^{2}|e_{i}^{u}\left(t\right)|+\sum_{i=1}^{2}\left|e_{i}^{v}\left(t\right)\right|,\\ V_{2}\left(t\right)=\sum_{i=1}^{2}\frac{1}{2k_{i}}{\left[X_{1}^{i}\left(t\right)\right]}^{2}+\sum_{i=1}^{2}\frac{1}{2m_{i}}{\left[X_{2}^{i}\left(t\right)\right]}^{2}+\sum_{i=1}^{2}\frac{1}{2l_{i}}{\left[X_{3}^{i}\left(t\right)\right]}^{2}+\sum_{i=1}^{2}\frac{1}{2n_{i}}{\left[X_{4}^{i}\left(t\right)\right]}^{2}+\sum_{i=1}^{2}\frac{1}{2q_{i}}{\left[X_{5}^{i}\left(t\right)\right]}^{2}, \end{array}$$

where $X_{1}^{i}\left(t\right)=d_{i}\left(t\right)-d_{i}$, $X_{2}^{i}\left(t\right)=\eta_{i}\left(t\right)-\eta_{i}$, $X_{3}^{i}\left(t\right)=p_{i}\left(t\right)-p_{i}$, $X_{4}^{i}\left(t\right)=\theta_{i}\left(t\right)-\theta_{i}$, $X_{5}^{i}\left(t\right)=w_{i}\left(t\right)-w_{i}$, $d_{i}$, $\eta_{i}$, $p_{i}$, $w_{i}$ and $\theta_{i}$ are constants to be determined later.

Now, construct a Lyapunov-like function as follows:

(12) $$\begin{array}{c} V\left(t\right)=V_{1}\left(t\right)+V_{2}\left(t\right). \end{array}$$

Based on Lemma 1, Lemma 2, one has

$$\begin{array}{cc} D^{\alpha }V\left(t\right) & \leq \sum_{i=1}^{2}sgn\left(e_{i}^{u}\left(t\right)\right)D^{\alpha }e_{i}^{u}\left(t\right)+\sum_{i=1}^{2}sgn\left(e_{i}^{v}\left(t\right)\right)D^{\alpha }e_{i}^{v}\left(t\right)+\sum_{i=1}^{2}\frac{1}{k_{i}}\left[d_{i}\left(t\right)-d_{i}\right]D^{\alpha }d_{i}\left(t\right)+\\ & \sum_{i=1}^{2}\frac{1}{m_{i}}\left[\eta_{i}\left(t\right)-\eta_{i}\right]D^{\alpha }\eta_{i}\left(t\right)+\sum_{i=1}^{2}\frac{1}{l_{i}}\left[p_{i}\left(t\right)-p_{i}\right]D^{\alpha }p_{i}\left(t\right)+\sum_{i=1}^{2}\frac{1}{n_{i}}\left[\theta_{i}\left(t\right)-\theta_{i}\right]D^{\alpha }\theta_{i}\left(t\right)+\\ & \sum_{i=1}^{2}\frac{1}{q_{i}}\left[w_{i}\left(t\right)-w_{i}\right]D^{\alpha }w_{i}\left(t\right). \end{array}$$

See the Appendix for the proof of $D^{\alpha }V\left(t\right)\leq -\zeta V_{1}\left(t\right)\leq 0$, where ζ is a positive constant.

From Definition 1 and (A1), one has

$$V\left(t\right)-V\left(t_{0}\right)=\frac{1}{\Gamma \left(\alpha \right)}\int_{t_{0}}^{t}{\left(t-s\right)}^{\alpha -1}D^{\alpha }V\left(s\right)\;ds\leq 0.$$

Therefore $V\left(t\right)\leq V\left(t_{0}\right)$, $t\geq t_{0}$. Then from (12), one knows that $e_{i}^{u}\left(t\right)$, $e_{i}^{v}\left(t\right)$, $d_{i}\left(t\right)$, $\eta_{i}\left(t\right)$, $p_{i}\left(t\right)$, $\theta_{i}\left(t\right)$ and $w_{i}\left(t\right)$ are bounded on $t\geq t_{0}$. Thus, one can obtain there exists a positive constant N>0 satisfying

(13) $$|D^{\alpha }V_{1}\left(t\right)|\leq N,\;t\geq t_{0}.$$

We declare that $\lim_{t\to \infty }V_{1}\left(t\right)=0$.

In [36], the authors have given the proof of $\lim_{t\to \infty }V_{1}\left(t\right)=0$ by contradiction. Thus, the drive system (6) and the response system (7) are globally asymptotically synchronized under the controller (11). This completes the proof. ☐

## 4. Numerical Simulations

In this section, some numerical simulations will be provided to demonstrate the main results.

Consider the drive FCVNN (6) with α=0.99, τ=0.01, $I_{1}\left(t\right)=2\left(\sin t-i\cos t\right)$, $I_{2}\left(t\right)=\;\cos(t+1)+3i\sin\left(t-1\right)$, $a_{1}=1$, $a_{2}=2.5$, $a_{3}=0.5$, $b_{11}=1+i$, $b_{12}=-1.5+2i$, $b_{21}=3.5+i$, $b_{22}=4.8-4.8i$, and

$$f\left(x_{i}\right)=\frac{1-e^{-u_{i}}}{1+e^{-u_{i}}}+i\frac{1}{1+e^{-v_{i}}},\;g\left(x_{i}\right)=\frac{1-e^{-v_{i}}}{1+e^{-v_{i}}}+i\frac{1}{1+e^{-u_{i}}},$$

where i=1,2,3. The response FCVNN (7) share the same parameters with (6).

It is easy to compute $\lambda_{i}^{RR}=0.5$, $\lambda_{i}^{II}=0.25$, $\lambda_{i}^{RI}=\lambda_{i}^{IR}=0$, $\mu_{i}^{RR}=\mu_{i}^{II}=0$, $\mu_{i}^{RI}=0.5$, $\mu_{i}^{IR}=0.25$. The initial conditions are taken as

(14) $$\left\{\begin{array}{c} x_{1}\left(s\right)=-2+1.5i,\;x_{2}\left(s\right)=-2+2i,\;x_{3}\left(s\right)=2-6i,\\ y_{1}\left(s\right)=-6-i,\;y_{2}\left(s\right)=-1-2.5i,\;y_{3}\left(s\right)=-5+2i, \end{array}\right.\;s\in \left[-1,0\right].$$

And let $\eta_{1}\left(0\right)=0.1$, $\eta_{2}\left(0\right)=0.1$, $d_{1}\left(0\right)=0.01$, $d_{2}\left(0\right)=0.01$, $p_{1}\left(0\right)=0.01$, $p_{2}\left(0\right)=0.01$, $\theta_{1}\left(0\right)=0.01$, $\theta_{2}\left(0\right)=0.01$, $w_{1}\left(0\right)=0.2$, $w_{2}\left(0\right)=0.3$, $k_{1}=0.2$, $k_{2}=0.04$, $m_{1}=0.2$, $m_{2}=0.01$, $l_{1}=0.05$, $l_{2}=0.02$, $n_{1}=0.01$, $n_{2}=0.04$, $q_{1}=0.3$, $q_{2}=0.05$, $\eta_{1}=2.5$, $\eta_{2}=1.5$, $d_{1}=1$, $d_{2}=3$, $p_{1}=1$, $p_{2}=3$, $\theta_{1}=5$, $\theta_{2}=2$, $w_{1}=3$, $w_{2}=2$. By calculation, one obtains

$$\begin{array}{l} a_{1}+d_{1}>0,\;a_{2}+d_{2}-|b_{21}^{R}|\lambda_{2}^{RR}-|b_{21}^{I}|\lambda_{2}^{IR}-|b_{21}^{R}|\lambda_{2}^{IR}-\left|b_{21}^{I}\right|\lambda_{2}^{RR}>0,\\ w_{1}-|b_{11}^{R}|\lambda_{3}^{RR}-|b_{11}^{I}|\lambda_{3}^{IR}-|b_{11}^{R}|\lambda_{3}^{IR}-\left|b_{11}^{I}\right|\lambda_{3}^{RR}>0,\\ w_{2}-|b_{11}^{R}|\lambda_{3}^{RI}-|b_{11}^{I}|\lambda_{3}^{II}-|b_{11}^{R}|\lambda_{3}^{II}-\left|b_{11}^{I}\right|\lambda_{3}^{RI}>0,\\ \eta_{1}-|b_{22}^{R}|\mu_{1}^{RR}-|b_{22}^{I}|\mu_{1}^{IR}-|b_{22}^{R}|\mu_{1}^{IR}-\left|b_{22}^{I}\right|\mu_{1}^{RR}>0,\\ \eta_{2}-|b_{12}^{R}|\mu_{2}^{RR}-|b_{12}^{I}|\mu_{2}^{IR}-|b_{12}^{R}|\mu_{2}^{IR}-\left|b_{12}^{I}\right|\mu_{2}^{RR}>0,\\ a_{1}+p_{1}>0,\;a_{2}+p_{2}-|b_{21}^{R}|\lambda_{2}^{RI}-|b_{21}^{I}|\lambda_{2}^{II}-|b_{21}^{R}|\lambda_{2}^{II}-\left|b_{21}^{I}\right|\lambda_{2}^{RI}>0,\\ \theta_{1}-|b_{22}^{R}|\mu_{1}^{RI}-|b_{22}^{I}|\mu_{1}^{II}-|b_{22}^{R}|\mu_{1}^{II}-\left|b_{22}^{I}\right|\mu_{1}^{RI}>0,\\ \theta_{2}-|b_{12}^{R}|\mu_{2}^{RI}-|b_{12}^{I}|\mu_{2}^{II}-|b_{12}^{R}|\mu_{2}^{II}-\left|b_{12}^{I}\right|\mu_{2}^{RI}>0. \end{array}$$

**Figure 1:**
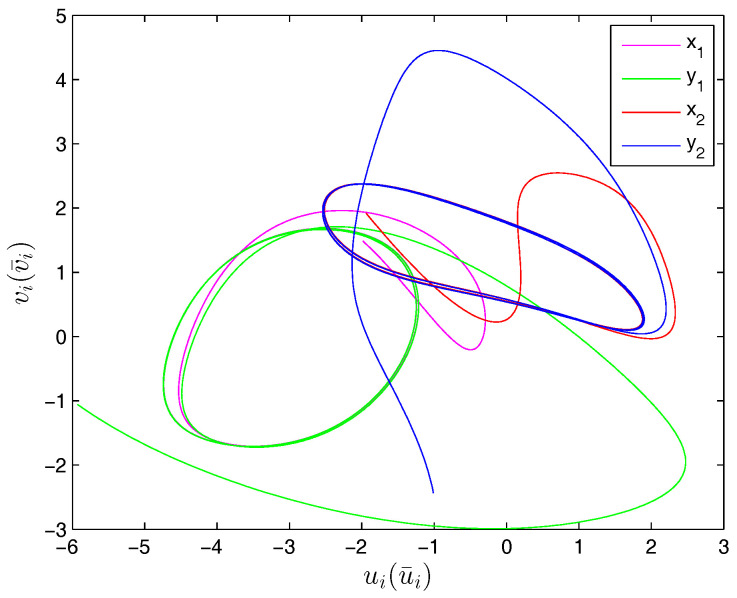
Time evolution of states $x_{1}$, $x_{2}$, $y_{1}$ and $y_{2}$ in 2-*D* plane.

**Figure 2:**
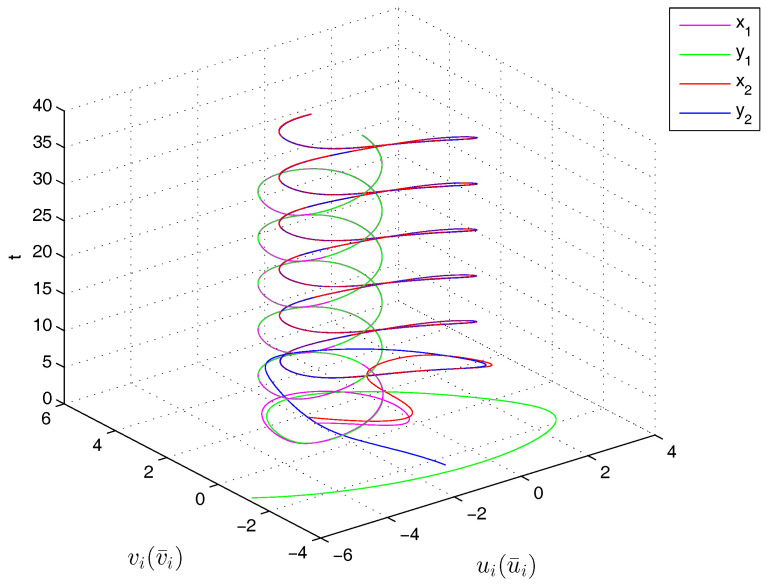
Time evolution of $x_{1}$, $x_{2}$, $y_{1}$ and $y_{2}$ in 3-*D* space.

**Figure 3:**
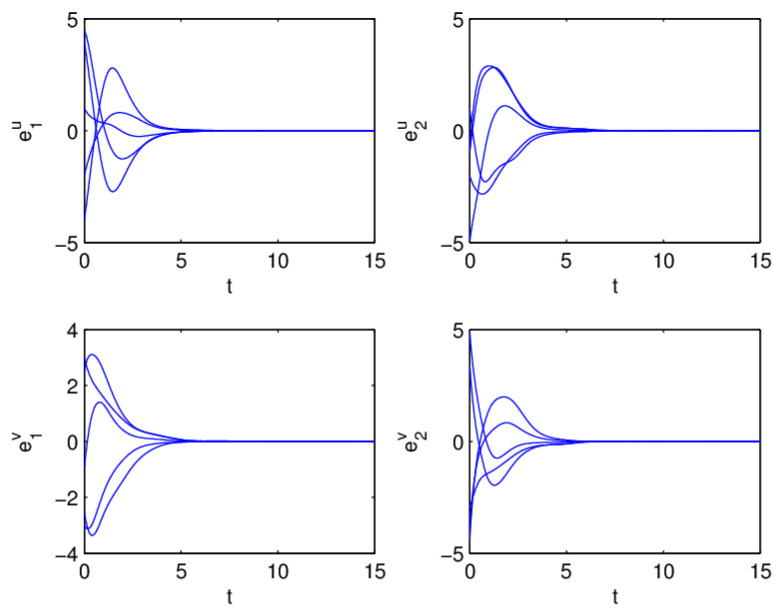
Synchronization errors $e_{i}\left(t\right)=y_{i}\left(t\right)-x_{i}\left(t\right)$ with five different initial values, i=1,2.

**Figure 4:**
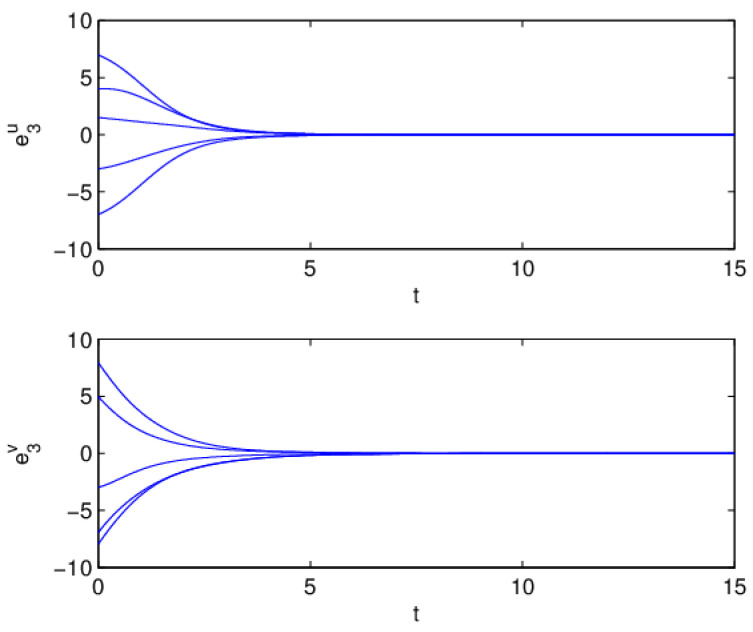
Synchronization errors $e_{3}^{u}\left(t\right)$ and $e_{3}^{v}\left(t\right)$ with five different initial values.

**Figure 5:**
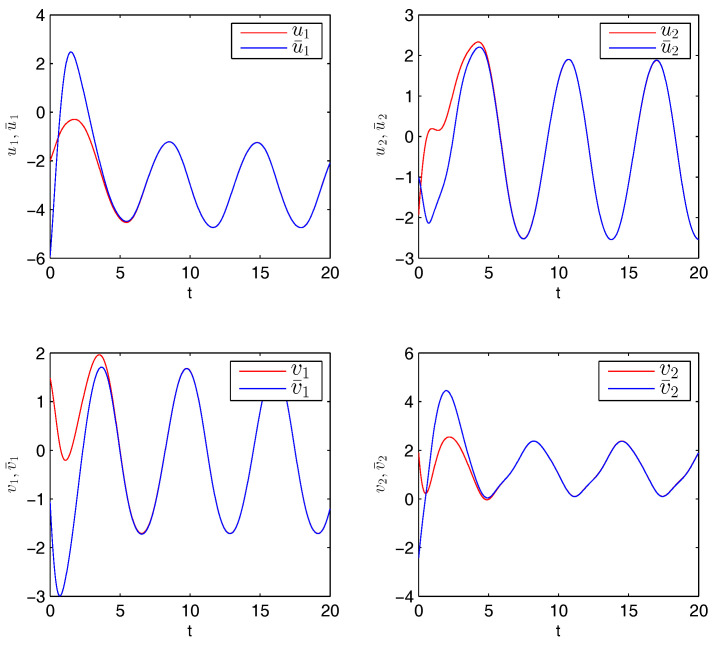
Time revolution of system (6) and (7) with controllers as Equation (11).

**Figure 6:**
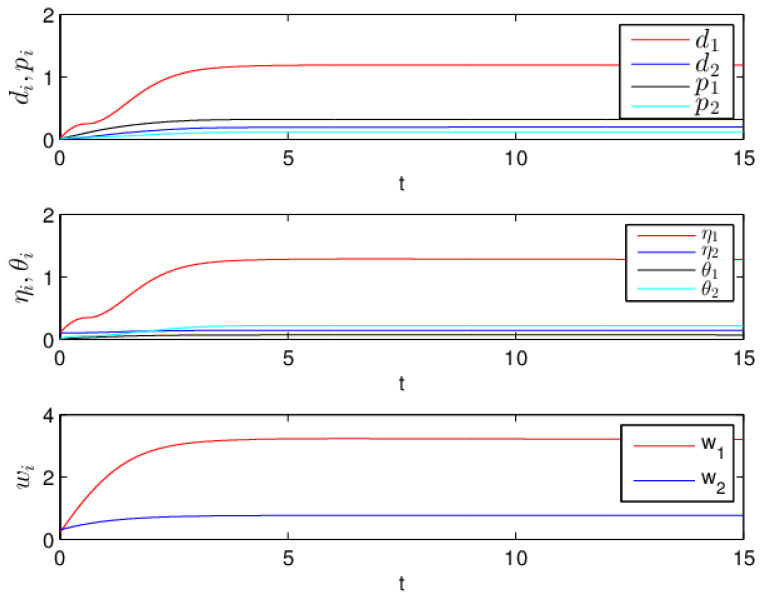
Time response of the feedback gains $d_{i}\left(t\right)$, $p_{i}\left(t\right)$, $\eta_{i}\left(t\right)$, $\theta_{i}\left(t\right)$ and $w_{i}\left(t\right)$.

Therefore, from Theorem 1, the drive system (6) and the response system (7) with the initial values (14) can achieve globally asymptotically synchronization under the controller (11). The curves of states $x_{1}$, $x_{2}$, $y_{1}$ and $y_{2}$ in 2-dimensional plane and 3-dimensional space when achieving synchronization are depicted in Figure 1 and Figure 2, respectively. Figure 3 shows the errors between $y_{i}$ and $x_{i}\left(i=1,2\right)$ with five different initial values. The errors of the introduced variables are plotted in Figure 4. Figure 5 shows the time revolution of real and imaginary parts of $x_{1}$, $x_{2}$, $y_{1}$ and $y_{2}$ with the controller (11), respectively. From simulation results in Figure 1, Figure 2, Figure 3, Figure 4 and Figure 5, it is clearly seen that the drive system (6) and the response system (7) can achieve synchronization. Figure 6 shows time response of the adaptive feedback gains $d_{i}\left(t\right)$, $p_{i}\left(t\right)$, $\eta_{i}\left(t\right)$, $\theta_{i}\left(t\right)$ and $w_{i}\left(t\right)\;\left(i=1,2\right)$.

## 5. Conclusions

In this paper, based on adaptive control and fractional-order Lyapunov-like function method, the information synchronization of drive-response FCVNNs with discrete and distributed delays has been studied. Due to the consideration of distributed delay, a new variable is defined to convert the FCVNN into a system with only discrete time delay. When systems (6) and (7) achieve information synchronization, the errors of the introduced variables tend to zero. The adaptive controller is designed in a elaborate way. Some sufficient conditions are developed to achieve the information synchronization. Numerical results show the effectiveness and correctness of the theoretical result.

## Appendix A

From (10), (11) and the above formula, one has

$$\begin{array}{cc} D^{\alpha }V\left(t\right) & \leq sgn\left(e_{1}^{u}\left(t\right)\right)\{-a_{1}e_{1}^{u}\left(t\right)+b_{11}^{R}\left[f^{R}\left({\bar{u}}_{3}\left(t\right),{\bar{v}}_{3}\left(t\right)\right)-f^{R}\left(u_{3}\left(t\right),v_{3}\left(t\right)\right)\right]-b_{11}^{I}[f^{I}\left({\bar{u}}_{3}\left(t\right),{\bar{v}}_{3}\left(t\right)\right)-\\ & f^{I}\left(u_{3}\left(t\right),v_{3}\left(t\right)\right)]+b_{12}^{R}\left[g^{R}\left({\bar{u}}_{2}\left(t-\tau \right),{\bar{v}}_{2}\left(t-\tau \right)\right)-g^{R}\left(u_{2}\left(t-\tau \right),v_{2}\left(t-\tau \right)\right)\right]-\\ & b_{12}^{I}\left[g^{I}\left({\bar{u}}_{2}\left(t-\tau \right),{\bar{v}}_{2}\left(t-\tau \right)\right)-g^{I}\left(u_{2}\left(t-\tau \right),v_{2}\left(t-\tau \right)\right)\right]-d_{1}\left(t\right)e_{1}^{u}\left(t\right)-sgn\left(e_{1}^{u}\left(t\right)\right)\eta_{1}\left(t\right)\left|e_{1}^{u}\left(t-\tau \right)\right|\\ & -w_{1}\left(t\right)e_{3}^{u}\left(t\right)\}+sgn\left(e_{2}^{u}\left(t\right)\right)\{-a_{2}e_{2}^{u}\left(t\right)+b_{21}^{R}\left[f^{R}\left({\bar{u}}_{2}\left(t\right),{\bar{v}}_{2}\left(t\right)\right)-f^{R}\left(u_{2}\left(t\right),v_{2}\left(t\right)\right)\right]-\\ & b_{21}^{I}\left[f^{I}\left({\bar{u}}_{2}\left(t\right),{\bar{v}}_{2}\left(t\right)\right)-f^{I}\left(u_{2}\left(t\right),v_{2}\left(t\right)\right)\right]+b_{22}^{R}\left[g^{R}\left({\bar{u}}_{1}\left(t-\tau \right),{\bar{v}}_{1}\left(t-\tau \right)\right)-g^{R}\left(u_{1}\left(t-\tau \right),v_{1}\left(t-\tau \right)\right)\right]\\ & -b_{22}^{I}\left[g^{I}\left({\bar{u}}_{1}\left(t-\tau \right),{\bar{v}}_{1}\left(t-\tau \right)\right)-g^{I}\left(u_{1}\left(t-\tau \right),v_{1}\left(t-\tau \right)\right)\right]-d_{2}\left(t\right)e_{2}^{u}\left(t\right)-\\ & sgn\left(e_{2}^{u}\left(t\right)\right)\eta_{2}\left(t\right)\left|e_{2}^{u}\left(t-\tau \right)\right|\}+sgn\left(e_{1}^{v}\left(t\right)\right)\{-a_{1}e_{1}^{v}\left(t\right)+b_{11}^{R}\left[f^{I}\left({\bar{u}}_{3}\left(t\right),{\bar{v}}_{3}\left(t\right)\right)-f^{I}\left(u_{3}\left(t\right),v_{3}\left(t\right)\right)\right]+\\ & b_{11}^{I}\left[f^{R}\left({\bar{u}}_{3}\left(t\right),{\bar{v}}_{3}\left(t\right)\right)-f^{R}\left(u_{3}\left(t\right),v_{3}\left(t\right)\right)\right]+b_{12}^{R}\left[g^{I}\left({\bar{u}}_{2}\left(t-\tau \right),{\bar{v}}_{2}\left(t-\tau \right)\right)-g^{I}\left(u_{2}\left(t-\tau \right),v_{2}\left(t-\tau \right)\right)\right]\\ & +b_{12}^{I}\left[g^{R}\left({\bar{u}}_{2}\left(t-\tau \right),{\bar{v}}_{2}\left(t-\tau \right)\right)-g^{R}\left(u_{2}\left(t-\tau \right),v_{2}\left(t-\tau \right)\right)\right]-p_{1}\left(t\right)e_{1}^{v}\left(t\right)-\\ & sgn\left(e_{1}^{v}\left(t\right)\right)\theta_{1}\left(t\right)|e_{1}^{v}\left(t-\tau \right)|-w_{2}\left(t\right)e_{3}^{v}\left(t\right)\}+sgn\left(e_{2}^{v}\left(t\right)\right)\{-a_{2}e_{2}^{v}\left(t\right)+b_{21}^{R}[f^{I}\left({\bar{u}}_{2}\left(t\right),{\bar{v}}_{2}\left(t\right)\right)-\\ & f^{I}\left(u_{2}\left(t\right),v_{2}\left(t\right)\right)]+b_{21}^{I}\left[f^{R}\left({\bar{u}}_{2}\left(t\right),{\bar{v}}_{2}\left(t\right)\right)-f^{R}\left(u_{2}\left(t\right),v_{2}\left(t\right)\right)\right]+b_{22}^{R}[g^{I}\left({\bar{u}}_{1}\left(t-\tau \right),{\bar{v}}_{1}\left(t-\tau \right)\right)-\\ & g^{I}\left(u_{1}\left(t-\tau \right),v_{1}\left(t-\tau \right)\right)]+b_{22}^{I}\left[g^{R}\left({\bar{u}}_{1}\left(t-\tau \right),{\bar{v}}_{1}\left(t-\tau \right)\right)-g^{R}\left(u_{1}\left(t-\tau \right),v_{1}\left(t-\tau \right)\right)\right]-p_{2}\left(t\right)e_{2}^{v}\left(t\right)\\ & -sgn\left(e_{2}^{v}\left(t\right)\right)\theta_{2}\left(t\right)|e_{2}^{v}\left(t-\tau \right)|\}+\sum_{i=1}^{2}\frac{1}{k_{i}}\left(d_{i}\left(t\right)-d_{i}\right)k_{i}\left|e_{i}^{u}\left(t\right)\right|+\\ & \sum_{i=1}^{2}\frac{1}{m_{i}}\left(\eta_{i}\left(t\right)-\eta_{i}\right)m_{i}|e_{i}^{u}\left(t-\tau \right)|+\sum_{i=1}^{2}\frac{1}{l_{i}}\left(p_{i}\left(t\right)-p_{i}\right)l_{i}\left|e_{i}^{v}\left(t\right)\right|+\\ & \sum_{i=1}^{2}\frac{1}{n_{i}}\left(\theta_{i}\left(t\right)-\theta_{i}\right)n_{i}|e_{i}^{v}\left(t-\tau \right)|+\frac{1}{q_{1}}\left(w_{1}\left(t\right)-w_{1}\right)q_{1}|e_{3}^{u}\left(t\right)|+\frac{1}{q_{2}}\left(w_{2}\left(t\right)-w_{2}\right)q_{2}\left|e_{3}^{v}\left(t\right)\right|\\ & \leq \{-a_{1}|e_{1}^{u}\left(t\right)|+|b_{11}^{R}\left|\right|f^{R}\left({\bar{u}}_{3}\left(t\right),{\bar{v}}_{3}\left(t\right)\right)-f^{R}\left(u_{3}\left(t\right),v_{3}\left(t\right)\right)\left|+\right|b_{11}^{I}\left|\right|f^{I}\left({\bar{u}}_{3}\left(t\right),{\bar{v}}_{3}\left(t\right)\right)-f^{I}\left(u_{3}\left(t\right),v_{3}\left(t\right)\right)|\\ & +|b_{12}^{R}\left|\right|g^{R}\left({\bar{u}}_{2}\left(t-\tau \right),{\bar{v}}_{2}\left(t-\tau \right)\right)-g^{R}\left(u_{2}\left(t-\tau \right),v_{2}\left(t-\tau \right)\right)\left|+\right|b_{12}^{I}\left|\right|g^{I}\left({\bar{u}}_{2}\left(t-\tau \right),{\bar{v}}_{2}\left(t-\tau \right)\right)-\\ & g^{I}\left(u_{2}\left(t-\tau \right),v_{2}\left(t-\tau \right)\right)|-d_{1}|e_{1}^{u}\left(t\right)|-\eta_{1}|e_{1}^{u}\left(t-\tau \right)|-w_{1}|e_{3}^{u}\left(t\right)|\}+\{-a_{2}\left|e_{2}^{u}\left(t\right)\right|+\\ & |b_{21}^{R}\left|\right|f^{R}\left({\bar{u}}_{2}\left(t\right),{\bar{v}}_{2}\left(t\right)\right)-f^{R}\left(u_{2}\left(t\right),v_{2}\left(t\right)\right)\left|+\right|b_{21}^{I}\left|\right|f^{I}\left({\bar{u}}_{2}\left(t\right),{\bar{v}}_{2}\left(t\right)\right)-f^{I}\left(u_{2}\left(t\right),v_{2}\left(t\right)\right)|+\\ & |b_{22}^{R}\left|\right|g^{R}\left({\bar{u}}_{1}\left(t-\tau \right),{\bar{v}}_{1}\left(t-\tau \right)\right)-g^{R}\left(u_{1}\left(t-\tau \right),v_{1}\left(t-\tau \right)\right)\left|+\right|b_{22}^{I}\left|\right|g^{I}\left({\bar{u}}_{1}\left(t-\tau \right),{\bar{v}}_{1}\left(t-\tau \right)\right)- \end{array}$$

$$\begin{array}{c} \;\;g^{I}\left(u_{1}\left(t-\tau \right),v_{1}\left(t-\tau \right)\right)|-d_{2}|e_{2}^{u}\left(t\right)|-\eta_{2}|e_{2}^{u}\left(t-\tau \right)|\}+\{-a_{1}|e_{1}^{v}\left(t\right)|+|b_{11}^{R}\left|\right|f^{I}\left({\bar{u}}_{3}\left(t\right),{\bar{v}}_{3}\left(t\right)\right)-\\ \;\;f^{I}\left(u_{3}\left(t\right),v_{3}\left(t\right)\right)\left|+\right|b_{11}^{I}\left|\right|f^{R}\left({\bar{u}}_{3}\left(t\right),{\bar{v}}_{3}\left(t\right)\right)-f^{R}\left(u_{3}\left(t\right),v_{3}\left(t\right)\right)\left|+\right|b_{12}^{R}\left|\right|g^{I}\left({\bar{u}}_{2}\left(t-\tau \right),{\bar{v}}_{2}\left(t-\tau \right)\right)-\\ \;\;g^{I}\left(u_{2}\left(t-\tau \right),v_{2}\left(t-\tau \right)\right)\left|+\right|b_{12}^{I}\left|\right|g^{R}\left({\bar{u}}_{2}\left(t-\tau \right),{\bar{v}}_{2}\left(t-\tau \right)\right)-g^{R}\left(u_{2}\left(t-\tau \right),v_{2}\left(t-\tau \right)\right)|-p_{1}\left|e_{1}^{v}\left(t\right)\right|-\\ \;\;\theta_{1}|e_{1}^{v}\left(t-\tau \right)|-w_{2}|e_{3}^{v}\left(t\right)|\}+\{-a_{2}|e_{2}^{v}\left(t\right)|+|b_{21}^{R}\left|\right|f^{I}\left({\bar{u}}_{2}\left(t\right),{\bar{v}}_{2}\left(t\right)\right)-f^{I}\left(u_{2}\left(t\right),v_{2}\left(t\right)\right)|+\\ \;\;|b_{21}^{I}\left|\right|f^{R}\left({\bar{u}}_{2}\left(t\right),{\bar{v}}_{2}\left(t\right)\right)-f^{R}\left(u_{2}\left(t\right),v_{2}\left(t\right)\right)\left|+\right|b_{22}^{R}\left|\right|g^{I}\left({\bar{u}}_{1}\left(t-\tau \right),{\bar{v}}_{1}\left(t-\tau \right)\right)-\\ \;\;g^{I}\left(u_{1}\left(t-\tau \right),v_{1}\left(t-\tau \right)\right)\left|+\right|b_{22}^{I}\left|\right|g^{R}\left({\bar{u}}_{1}\left(t-\tau \right),{\bar{v}}_{1}\left(t-\tau \right)\right)-g^{R}\left(u_{1}\left(t-\tau \right),v_{1}\left(t-\tau \right)\right)|-\\ \;\;p_{2}|e_{2}^{v}\left(t\right)|-\theta_{2}\left|e_{2}^{v}\left(t-\tau \right)\right|\}, \end{array}$$

From Assumptions 1 and 2, one has

$$\begin{array}{cc} D^{\alpha }V\left(t\right) & \leq \{-a_{1}|e_{1}^{u}\left(t\right)|+|b_{11}^{R}|[\lambda_{3}^{RR}|e_{3}^{u}\left(t\right)|+\lambda_{3}^{RI}|e_{3}^{v}\left(t\right)|]+|b_{11}^{I}|[\lambda_{3}^{IR}|e_{3}^{u}\left(t\right)|+\lambda_{3}^{II}|e_{3}^{v}\left(t\right)\left|\right]+\\ & |b_{12}^{R}|[\mu_{2}^{RR}|e_{2}^{u}\left(t-\tau \right)|+\mu_{2}^{RI}|e_{2}^{v}\left(t-\tau \right)|]+|b_{12}^{I}|[\mu_{2}^{IR}|e_{2}^{u}\left(t-\tau \right)|+\mu_{2}^{II}|e_{2}^{v}\left(t-\tau \right)|]-d_{1}\left|e_{1}^{u}\left(t\right)\right|-\\ & \eta_{1}|e_{1}^{u}\left(t-\tau \right)|-w_{1}|e_{3}^{u}\left(t\right)|\}+\{-a_{2}|e_{2}^{u}\left(t\right)|+|b_{21}^{R}|[\lambda_{2}^{RR}|e_{2}^{u}\left(t\right)|+\lambda_{2}^{RI}|e_{2}^{v}\left(t\right)|]+|b_{21}^{I}|[\lambda_{2}^{IR}\left|e_{2}^{u}\left(t\right)\right|+\\ & \lambda_{2}^{II}|e_{2}^{v}\left(t\right)|]+|b_{22}^{R}|[\mu_{1}^{RR}|e_{1}^{u}\left(t-\tau \right)|+\mu_{1}^{RI}|e_{1}^{v}\left(t-\tau \right)|]+|b_{22}^{I}|[\mu_{1}^{IR}|e_{1}^{u}\left(t-\tau \right)|+\mu_{1}^{II}|e_{1}^{v}\left(t-\tau \right)\left|\right]-\\ & d_{2}|e_{2}^{u}\left(t\right)|-\eta_{2}|e_{2}^{u}\left(t-\tau \right)|\}+\{-a_{1}|e_{1}^{v}\left(t\right)|+|b_{11}^{R}|[\lambda_{3}^{IR}|e_{3}^{u}\left(t\right)|+\lambda_{3}^{II}|e_{3}^{v}\left(t\right)|]+|b_{11}^{I}|[\lambda_{3}^{RR}\left|e_{3}^{u}\left(t\right)\right|+\\ & \lambda_{3}^{RI}|e_{3}^{v}\left(t\right)|]+|b_{12}^{R}|[\mu_{2}^{IR}|e_{2}^{u}\left(t-\tau \right)|+\mu_{2}^{II}|e_{2}^{v}\left(t-\tau \right)|]+|b_{12}^{I}|[\mu_{2}^{RR}|e_{2}^{u}\left(t-\tau \right)|+\mu_{2}^{RI}|e_{2}^{v}\left(t-\tau \right)\left|\right]-\\ & p_{1}|e_{1}^{v}\left(t\right)|-\theta_{1}|e_{1}^{v}\left(t-\tau \right)|-w_{2}|e_{3}^{v}\left(t\right)|\}+\{-a_{2}|e_{2}^{v}\left(t\right)|+|b_{21}^{R}|[\lambda_{2}^{IR}|e_{2}^{u}\left(t\right)|+\lambda_{2}^{II}|e_{2}^{v}\left(t\right)\left|\right]+\\ & |b_{21}^{I}|[\lambda_{2}^{RR}|e_{2}^{u}\left(t\right)|+\lambda_{2}^{RI}|e_{2}^{v}\left(t\right)|]+|b_{22}^{R}|[\mu_{1}^{IR}|e_{1}^{u}\left(t-\tau \right)|+\mu_{1}^{II}|e_{1}^{v}\left(t-\tau \right)|]+|b_{22}^{I}|[\mu_{1}^{RR}\left|e_{1}^{u}\left(t-\tau \right)\right|+\\ & \mu_{1}^{RI}|e_{1}^{v}\left(t-\tau \right)|]-p_{2}|e_{2}^{v}\left(t\right)|-\theta_{2}\left|e_{2}^{v}\left(t-\tau \right)\right|\}\\ & =[\left(-a_{1}-d_{1}\right)|e_{1}^{u}\left(t\right)|+(-a_{2}-d_{2}+|b_{21}^{R}|\lambda_{2}^{RR}+|b_{21}^{I}|\lambda_{2}^{IR}+|b_{21}^{R}|\lambda_{2}^{IR}+|b_{21}^{I}|\lambda_{2}^{RR})|e_{2}^{u}\left(t\right)|+(-w_{1}+\\ & |b_{11}^{R}|\lambda_{3}^{RR}+|b_{11}^{I}|\lambda_{3}^{IR}+|b_{11}^{R}|\lambda_{3}^{IR}+|b_{11}^{I}|\lambda_{3}^{RR})|e_{3}^{u}\left(t\right)|+(-\eta_{1}+|b_{22}^{R}|\mu_{1}^{RR}+|b_{22}^{I}|\mu_{1}^{IR}+\left|b_{22}^{R}\right|\mu_{1}^{IR}+\\ & |b_{22}^{I}|\mu_{1}^{RR})|e_{1}^{u}\left(t-\tau \right)|+(-\eta_{2}+|b_{12}^{R}|\mu_{2}^{RR}+|b_{12}^{I}|\mu_{2}^{IR}+|b_{12}^{R}|\mu_{2}^{IR}+|b_{12}^{I}|\mu_{2}^{RR})|e_{2}^{u}\left(t-\tau \right)|]+\\ & [\left(-a_{1}-p_{1}\right)|e_{1}^{v}\left(t\right)|+(-a_{2}-p_{2}+|b_{21}^{R}|\lambda_{2}^{RI}+|b_{21}^{I}|\lambda_{2}^{II}+|b_{21}^{R}|\lambda_{2}^{II}+|b_{21}^{I}|\lambda_{2}^{RI})|e_{2}^{v}\left(t\right)|+(-w_{2}+\\ & |b_{11}^{R}|\lambda_{3}^{RI}+|b_{11}^{I}|\lambda_{3}^{II}+|b_{11}^{R}|\lambda_{3}^{II}+|b_{11}^{I}|\lambda_{3}^{RI})|e_{3}^{v}\left(t\right)|+(-\theta_{1}+|b_{22}^{R}|\mu_{1}^{RI}+|b_{22}^{I}|\mu_{1}^{II}+\left|b_{22}^{R}\right|\mu_{1}^{II}+\\ & |b_{22}^{I}|\mu_{1}^{RI})|e_{1}^{v}\left(t-\tau \right)|+(-\theta_{2}+|b_{12}^{R}|\mu_{2}^{RI}+|b_{12}^{I}|\mu_{2}^{II}+|b_{12}^{R}|\mu_{2}^{II}+|b_{12}^{I}|\mu_{2}^{RI})|e_{2}^{v}\left(t-\tau \right)|]. \end{array}$$

One can subtly choose $d_{i}$, $\eta_{i}$, $p_{i}$, $w_{i}$ and $\theta_{i}$ such that

$a_{1}+d_{1}>0,\;a_{2}+d_{2}-|b_{21}^{R}|\lambda_{2}^{RR}-|b_{21}^{I}|\lambda_{2}^{IR}-|b_{21}^{R}|\lambda_{2}^{IR}-\left|b_{21}^{I}\right|\lambda_{2}^{RR}>0,$
$w_{1}-|b_{11}^{R}|\lambda_{3}^{RR}-|b_{11}^{I}|\lambda_{3}^{IR}-|b_{11}^{R}|\lambda_{3}^{IR}-\left|b_{11}^{I}\right|\lambda_{3}^{RR}>0,$
$\eta_{1}-|b_{22}^{R}|\mu_{1}^{RR}-|b_{22}^{I}|\mu_{1}^{IR}-|b_{22}^{R}|\mu_{1}^{IR}-\left|b_{22}^{I}\right|\mu_{1}^{RR}>0,$
$\eta_{2}-|b_{12}^{R}|\mu_{2}^{RR}-|b_{12}^{I}|\mu_{2}^{IR}-|b_{12}^{R}|\mu_{2}^{IR}-\left|b_{12}^{I}\right|\mu_{2}^{RR}>0,$
$a_{1}+p_{1}>0,\;a_{2}+p_{2}-|b_{21}^{R}|\lambda_{2}^{RI}-|b_{21}^{I}|\lambda_{2}^{II}-|b_{21}^{R}|\lambda_{2}^{II}-\left|b_{21}^{I}\right|\lambda_{2}^{RI}>0,$
$w_{2}-|b_{11}^{R}|\lambda_{3}^{RI}-|b_{11}^{I}|\lambda_{3}^{II}-|b_{11}^{R}|\lambda_{3}^{II}-\left|b_{11}^{I}\right|\lambda_{3}^{RI}>0,$
$\theta_{1}-|b_{22}^{R}|\mu_{1}^{RI}-|b_{22}^{I}|\mu_{1}^{II}-|b_{22}^{R}|\mu_{1}^{II}-\left|b_{22}^{I}\right|\mu_{1}^{RI}>0,$
$\theta_{2}-|b_{12}^{R}|\mu_{2}^{RI}-|b_{12}^{I}|\mu_{2}^{II}-|b_{12}^{R}|\mu_{2}^{II}-\left|b_{12}^{I}\right|\mu_{2}^{RI}>0.$

Let

$$\begin{array}{c} \zeta_{1}=min\left\{a_{1}+d_{1},\;a_{2}+d_{2}-|b_{21}^{R}|\lambda_{2}^{RR}-|b_{21}^{I}|\lambda_{2}^{IR}-|b_{21}^{R}|\lambda_{2}^{IR}-|b_{21}^{I}|\lambda_{2}^{RR},\;w_{1}-|b_{11}^{R}|\lambda_{3}^{RR}-\left|b_{11}^{I}\right|\lambda_{3}^{IR}-\right.\\ \;\left.|b_{11}^{R}|\lambda_{3}^{IR}-\left|b_{11}^{I}\right|\lambda_{3}^{RR}\right\},\\ \zeta_{2}=min\left\{a_{1}+p_{1},\;a_{2}+p_{2}-|b_{21}^{R}|\lambda_{2}^{RI}-|b_{21}^{I}|\lambda_{2}^{II}-|b_{21}^{R}|\lambda_{2}^{II}-|b_{21}^{I}|\lambda_{2}^{RI},\;w_{2}-|b_{11}^{R}|\lambda_{3}^{RI}-\left|b_{11}^{I}\right|\lambda_{3}^{II}-\right.\\ \;\left.|b_{11}^{R}|\lambda_{3}^{II}-\left|b_{11}^{I}\right|\lambda_{3}^{RI}\right\},\\ \gamma_{1}=min\left\{\eta_{1}-|b_{22}^{R}|\mu_{1}^{RR}-|b_{22}^{I}|\mu_{1}^{IR}-|b_{22}^{R}|\mu_{1}^{IR}-|b_{22}^{I}|\mu_{1}^{RR},\;\eta_{2}-|b_{12}^{R}|\mu_{2}^{RR}-|b_{12}^{I}|\mu_{2}^{IR}-\left|b_{12}^{R}\right|\mu_{2}^{IR}-\right.\\ \;\left.|b_{12}^{I}|\mu_{2}^{RR}\right\}, \end{array}$$

$$\begin{array}{c} \gamma_{2}=min\left\{\theta_{1}-|b_{22}^{R}|\mu_{1}^{RI}-|b_{22}^{I}|\mu_{1}^{II}-|b_{22}^{R}|\mu_{1}^{II}-|b_{22}^{I}|\mu_{1}^{RI},\;\theta_{2}-|b_{12}^{R}|\mu_{2}^{RI}-|b_{12}^{I}|\mu_{2}^{II}-\left|b_{12}^{R}\right|\mu_{2}^{II}-\right.\\ \;\left.|b_{12}^{I}|\mu_{2}^{RI}\right\}. \end{array}$$

Then, one can obtain

(A1) $$\begin{array}{cc} D^{\alpha }V\left(t\right) & \leq -\zeta_{1}\sum_{i=1}^{3}|e_{i}^{u}\left(t\right)|-\gamma_{1}\sum_{i=1}^{2}|e_{i}^{u}\left(t-\tau \right)|-\zeta_{2}\sum_{i=1}^{3}|e_{i}^{v}\left(t\right)|-\gamma_{2}\sum_{i=1}^{2}\left|e_{i}^{v}\left(t-\tau \right)\right|\\ & \leq -\zeta_{1}\sum_{i=1}^{3}|e_{i}^{u}\left(t\right)|-\zeta_{2}\sum_{i=1}^{3}\left|e_{i}^{v}\left(t\right)\right|\\ & \leq -\zeta V_{1}\left(t\right)\leq 0, \end{array}$$

where $\zeta =min\{\zeta_{1},\;\zeta_{2}\}>0$.
